# A Remarkable Case

**Published:** 1858-04

**Authors:** 


### A Remarkable Case.

In a discussion upon Hysteria, in the *Sóciete de Médecine du departement de la Seine*, a few weeks ago. M. Duparcque, reported the following case: "A young lady, aged 18 years was, in consequence of a rupture with her affianced, attacked with Hysteria. Her symptoms, at first, were such as are usually observed in this disease; a sensation as if a ball ascended from the hypogastric region to the stomach and pharynx, with convulsions, constituted the most prominent. Later, the convulsions alternated with the greatest variety of symptoms and accidents, both general and local, often presenting the appearance of the most acute diseases. She was one evening attacked with the cataleptic form of convulsions, and soon became cold; the whole muscular system was relaxed; complete insensibility; the circulation appeared to be completely arrested. This state continued, and so complete was the suspension of all the signs of life at the regular visit the next morning, that the Physician in attendance at the hospital Benjoin, pronounced her dead. But having examined the patient myself, I thought that I could hear indistinctly, feeble and irregular pulsation of the heart, and as there was some warmth about the chest, I determined to make some experiments before I sent the body to the dead room. A Compress doubled several times upon itself was dipped in boiling water and suddenly placed upon the epigastric region. There was an immediate contraction of the abdominal and thoracic walls, with an incomplete inspiration. This was the only effect from the first application. The second application was followed by a more perfect inspiration, movement, and so on. The patient soon became conscious, and in a short time was restored to health.

